# Bioprospecting the African Renaissance: The new value of *muthi *in South Africa

**DOI:** 10.1186/1746-4269-4-9

**Published:** 2008-03-27

**Authors:** Hanspeter CW Reihling

**Affiliations:** 1 Institute for Social Anthropology, Freie Universität Berlin, Landoltweg 9-11, 14195 Berlin, Germany; 2 African Gender Institute, University of Cape Town, All Africa House, Middle Campus, Rondebosch 7700, South Africa

## Abstract

This article gives an overview of anthropological research on bioprospecting in general and of available literature related to bioprospecting particularly in South Africa. It points out how new insights on value regimes concerning plant-based medicines may be gained through further research and is meant to contribute to a critical discussion about the ethics of Access and Benefit Sharing (ABS). In South Africa, traditional healers, plant gatherers, petty traders, researchers and private investors are assembled around the issues of standardization and commercialization of knowledge about plants. This coincides with a nation-building project which promotes the revitalization of local knowledge within the so called African Renaissance. A social science analysis of the transformation of so called Traditional Medicine (TM) may shed light onto this renaissance by tracing social arenas in which different regimes of value are brought into conflict. When medicinal plants turn into assets in a national and global economy, they seem to be manipulated and transformed in relation to their capacity to promote health, their market value, and their potential to construct new ethics of development. In this context, the translation of socially and culturally situated local knowledge about *muthi *into global pharmaceuticals creates new forms of agency as well as new power differentials between the different actors involved.

## Biovalue in the African Renaissance

A growing demand for herbal medicines in North America and Europe as well as epidemics like Malaria, Tuberculosis and HIV/AIDS in Sub-Sahara African have created an expanding market for indigenous herbal medicines. Traditional healers, plant gatherers, petty traders, research institutions and private corporations have assembled around the issues of standardization and commercialization of local knowledge about plants. In South Africa this coincides with a nation-building project, which promotes the revitalization of local knowledge in the so called "African Renaissance" [1]. The idea of such renaissance is as old as the Pan-Africanist movement and its struggle for a regeneration of the continent after slavery and colonialism [2]. More recently, it was proclaimed by Thabo Mbeki's presidential campaigns as synonymous to a "second liberation struggle" for self-discovery, democratization and economic development. There is reason to assume that the transformation of so called Traditional Medicine (TM) sheds light onto this renaissance by indicating how different regimes of value are brought into conflict. Indeed, the translation of socially and culturally situated local knowledge into global pharmaceuticals creates new forms of agency as well as new power differentials between corporate investors, scientists, plant gatherers, and traditional healers. The present article alludes to the transformation of TM in South Africa by giving an overview of key moments in bioprospecting, the "exploration of biodiversity for commercially valuable genetic and biochemical resources" [3]. It will thereby trace social arenas in which plant based medicines have been assigned biovalue.

Catherine Waldby first introduced the concept of biovalue to designate the relationship between the promotion of health, the production of capital and the question of ethics in the context of human tissue and stem cell research [4]. It is generated through the manipulation of biological processes in the laboratory and yields therapeutic as well as monetary revenue. Furthermore, as emphasized by Novas and Rose it is simultaneously commercial and ethical [5]. In the present review, the notion of biovalue will depict the assignment of value to genetic material derived from indigenous medical plants. Although plants markedly differ from human stem cells similar dynamics seem to be at stake. Following Waldby, we are able to discern at least three main points of relevance to bioprospecting in the African Renaissance, emerging from a review of existing policy papers, legal frameworks, and academic research. When medicinal plants turn into assets in a national and global economy they become manipulated and transformed in relation to their capacity to promote health, their market value, and their potential to construct a new ethics of development. Before addressing these three aspects, I will first of all point to some anthropological work relevant for future studies on bioprospecting in society. I then give an overview of literature on bioprospecting within Social and Cultural Anthropology. Finally, the paper will be point out how new insights on value regimes on plant-based medicines may be gained through further research.

## Medical Pluralisms, Technology and Science

The process of enlightenment, globalization, modernity or post-modernity has been characterized by accelerating social change in the face of ambiguity, fragmentation and a quest for new certainties. Human experience becomes tangled by anonymous flows of capital, technologies, goods, people, images and ideas that may restrain at the same time as they may expand subjects' agency [6]. While there is a growing number of studies about the effects of this process at large, few concentrate on the transformation of plant-based traditional medicines and even less do so in Africa. Teleological assumptions made by Modernization Theory have been challenged by research in Medical Anthropology. Accordingly, information and education based on "scientific facts" do not automatically result in a replacement of so called "traditional beliefs". While some local medical treatments vanish over time others may be transformed, revitalized or reinvented [7]. Furthermore, it has been documented how subjects choose therapies and etiology eclectically, thereby generating new systems of signification [8-10]. In this sense, the impact of biomedical science and technology results in medical pluralisms and the hybridization of medical practice.

If this is the case with contemporary African medical systems, it is vital to analyze how the appropriation of new practices takes place among the different actors involved and what new significations and meanings are produced by medical pluralism. However, biomedicine is not just another link in a harmonic alignment of health care providers. Dominant discourses based on cosmopolitan notions of health introduced by international development agencies, multinational corporations and state institutions may well conflict with the ones adopted by rural plant collectors, petty traders or traditional healers. It has been argued that in order to reach beyond a mere documentation of medical pluralisms, the process of signification must be deciphered by a thorough analysis of power relations [11]. These become particularly salient in the field of property rights. The Anthropology of Law has long addressed cultural variation in property regimes [12,13], trends towards flexible dispute resolution instead of rights based decision-making [14,15] and the local appropriation of legal forms in multiple settings [16].

These studies have hardly been linked to research on bioprospecting in society and on the role of the biosciences in the negotiation of property rights in developing countries. It has in fact long been argued that science in general assumes a hegemonic position through the construction of its object. Theorizing in the anthropology and sociology of knowledge emphasizes that the externalized products of human activity attain the character of objectivity through a dialectical relationship between humans and the social world [17-19]. Ethnography has shown that there is no straightforward trajectory from laboratory research and the discovery of so called facts to medical technologies and their rational implementation in the social field [20-23]. Beyond the physical level of biomedical disease, the process of signification is tied to the specific interests of the actors involved. Calling for a suspension of a conceptual nature/culture divide ethnographic studies of laboratory practice contend that artifacts themselves are involved in the construction of heterogeneous actor-networks of humans and non-human "actants" [20,24,25].

On the other hand, anthropological analysis of medicine, science and technology also went beyond epistemological issues and analyzed modes of commodification of life and the emergence of new forms of biopolitical government in the field of genetic resources derived from animals and humans [26-28]. Frequently, the artifacts of research and development are legitimated and disseminated along powerful global allegiances and social networks rather than being product of neutral reasoning based on natural cause and effect. In this context, the ethnographic study of pharmaceuticals and their biographies has become a promising new field of inquiry that, however, has hardly targeted herbal medicines [29-31]. Future research will have to include more inquiries into the enclosure of knowledge for the purpose of drug development concerned with genetic or biochemical resources derived from indigenous medical plants. Some relevant examples of anthropological research done in this field are particularly concerned with bioprospecting.

## Bioprospecting and "Biopiracy" in International Perspective

To date ethnographic studies concerning the process of bioprospecting have primarily focused on the Americas where major bioprospecting agreements were established between the U.S.-based International Cooperative Biodiversity Group (ICBG) and Latin American universities from the early 1990s onwards. In this context, some anthropologists started to suggest that the inclusion of local knowledge into national and international patent law is incompatible with local cosmologies and may ultimately inflict "symbolic violence" upon indigenous cultures [32,33]. Others have emphasized that these communities are not only passive victims of globalization but rather selectively appropriate cosmopolitan discourses about intellectual property rights for the pursuit of their own interest [34].

It has also been pointed out that the struggle for indigenous rights can even have racist undertones that for the sake of ethno-national endeavors, depict culture as static and unchanging [35]. Such essentialist understandings often result in claims to the "copyright of culture". For Brown, the enclosure of knowledge however, bears the risk of putting an end to science, human progress and even democracy [36]. In another worst-case scenario, bioprospecting could contribute to conflict between different ethnic groups or even nation states that claim ownership to a genetic resource as well as to internal social disintegration of communities [37]. The latter seems to be true especially when certain factions are externally appointed to be representative of an imagined community although they do not reflect the opinion of the majority of people concerned [36,38]. The role of NGOs in this process may vary from generating exaggerated expectations about potential benefits through commercialization of IK to an emphasis on its devastating effects. So-called non-profit organizations may even foreclose rights recognition by mediating the interest of multinational corporations [39]. NGOs can nevertheless bring entire bioprospecting programs to halt through accusations of biopiracy or theft of IK. This may be the case even though local resource providers haven't given their mandate to do so [40]. With regard to this problem, it has been claimed that the governance of potentially collaborating indigenous societies is the key to successful benefit-sharing and must therefore be strengthened through capable authorizing institutions as well as a "culture of responsible research" [41].

Critics within anthropology have questioned the notion of biopiracy as non-contractual collection and use of resources as a useful analytical category for it is itself based on an understanding of authorship that underlies the very expropriation of indigenous resources by powerful global actors. From this perspective, benefit-sharing agreements contribute to the construction of bounded communities. Through appropriation from the public domain intellectual property is extended by "closing" agricultural commons whose origin in reality is ambiguous [42,43]. Cori Hayden's analysis of a bilateral benefit-sharing agreement between the U.S. and Mexico suggests that bio-artifacts contain, reproduce, and represent people's interests, thereby shaping social relationships for each plant collected [44]. In this sense, bioprospecting makes "nature go public" since it generates new audiences for scientific "facts" among private investors and local communities alike. The designation of benefit-recipients whether scientists or local interlocutors whom they are to recruit, creates incentives "to value their resources in ways imagined by project founders and architects" [44,61]. At stake are new forms of governance that produce human subjectivities and natural objects which conceptualize themselves or become conceptualized by idioms of the neoliberal market.

Medicinal plants are not only passive objects of scientific reason; from the perspective of Actor-Network-Theory mentioned above, they attract and connect different human and non-human actors. Through bioprospecting in society the emergent networks are not only constructed in relation to scientific knowledge production, but also in relation to prospective plant producers, private investors, and holders of local knowledge about plants. In this context we can concur with Franklin in arguing that bioprospecting draws nature and its so-called stakeholders into globally emergent forms of biocapital [45]. As pointed out by Sunder Rajan biocapital is a system of exchanges in the life-sciences that leads to an "implosion" of epistemology and political economy. It generates a "hype" around potential medicines that attracts venture capital and creates a speculative marketplace [46]. As such, it is not necessarily based on the actual needs of patients but on the anticipation of future consumers who eventually, may generate high profits. Before turning to the global marketplace created through bioprospecting, I will first of all address its relevance for the promotion of health in South Africa more broadly.

## Bioprospecting and the Promotion of Health

The transformation of indigenous medical plants has been recently seen as prerequisite for effective health promotion including the reduction of poverty. Whereas under colonialism, health and African tradition were seen as rather contradictory, today there is a revived interest in the way in which so-called Indigenous Knowledge Systems (IKS) can be made applicable for public health. The World Bank's Indigenous Knowledge Program defines IKS as "problem solving strategies for local communities" to achieve the Millennium Development Goals, to promote the sustainable management of resources, to enhance health care and to fight HIV/AIDS [47]. The World Health Organization has a particular interest in Traditional Medicine, the evaluation of its safety and efficacy and its integration into national healthcare systems [48]. The emphasis on local knowledge among international development organizations partly stems from past experience in ineffective top-down driven technical interventions and a general move toward a discourse on "empowerment" and "participation" [49]. The latter have been promoted in view of reducing costs for health care in resource poor settings by transferring more responsibility to the target population itself. The extent to which the search for commercially valuable genetic resources in Southern Africa is tied to new modes of biopolitical government through public health has not yet been addressed through social science research. Indeed, the search for indigenous herbal medicines in South Africa seems to be part of a larger nation-building project called African Renaissance which implicitly draws upon local knowledge, the life-sciences, and the counties' rich biodiversity.

Lund argues that the architects of the new South Africa draw on a long-standing tradition of using biological analogies to interpret social phenomena and to promote national causes. After Apartheid the government took recourse to rhetoric around "healing the nation" using metaphors previously used in colonial and apartheid discourse [50]. Currently, the promotion of South Africa's vitality through bioprospecting takes place within a larger project of self-discovery and the revitalization of African culture coupled with an investment into the life-sciences. President Mbeki, in his well-known African Renaissance statement, emphasizes that "the colonizers sought to enslave the African mind and to destroy the African soul", therefore "the beginning of our rebirth as a continent must be our own rediscovery of our soul" [51]. Since then, much effort has been put into stimulating the contribution of IKS to health and development. The South African IKS policy aims at promoting a positive African identity by the affirmation of African cultural values and the development of services provided by holders of IK, especially in the field of Traditional Medicine. It further maintains that the role of indigenous knowledge in the creation of economic wealth must be strengthened and indigenous knowledge should be "used together with modern biotechnology in the pharmaceutical and other sectors to increase the rate of innovation" [52]. Considering South Africa's rich biodiversity, the connection between a "healthy nation" and biology is metaphoric as it is material. Roughly, 700 plant species are used and traded for medical purpose outside the formal biomedical system [53]. The local knowledge, including notions of health and healing, attached to them is currently being transformed.

In South Africa publicly funded research institutions have a marked interest in the commercialization of plant-based medicines purified of cultural and social derivates. All major South African universities have botany or biochemistry departments that engage in bioprospecting activities concerning TM; the same is true for large research institutions like the Medical Research Council (MRC) or the Council for Scientific and Industrial Research (CSIR). It is hoped that prospecting and the commercial cultivation of medical plants will yield new employment opportunities at the rural level and cost-effective drugs against regionally prevalent diseases [54]. In this context, the promotion of health seems to be tied to the enclosure of knowledge about plants. This enclosure however equates local knowledge with information relevant for bioprospecting. The National Patents Act was amended to include a definition of traditional knowledge as "knowledge that an indigenous community has regarding the use of a biological or genetic resource". Indeed, the cosmopolitan language of the life-sciences favors certain aspects being immediately compatible with analyzing plant based medicines in the laboratory, while neglecting others that may still be meaningful to local producers, distributors, patients and healers [7]. In South Africa approximately 350.000 Traditional Healers offer medicinal plants to 60–80% of the population [55]. Wreford argues that while healers are quite interested in partnerships and associations with biomedicine, the enthusiasm is hardly reciprocated. They are expected to adapt to biomedicine in a "unidirectional project" [56].

Indeed, mutual understanding between traditional healers, state officials, and scientists seems to be rather limited [57]. Once highlighted by Ngubane [58], the distinction between herbalist or *inyanga *and diviner or *sangoma *is now widely endorsed by the state. For research institutions *inyangas *are the main representatives of local knowledge due to their assumed compatibility with Western epistemology. Although patients hardly distinguish them from *sangomas*, the latter have been confined to the realm of religion and belief [57]. Such categorization seems to deceptive considering the complex interrelationships between the physical, the social and the spiritual world, established through African healers' diagnosis and treatment [10]. This becomes salient in the concept of *muthi*, which derives from the Nguni root -*thi *and signifies tree. It refers to a substance or mixture of different substances primarily derived from plant material and processed in a way either to achieve positive effects of healing, cleansing and protection from evil forces or negative ones of witchcraft, misfortune and ill-health. A study of Xhosa chemists in Eastern Cape Province by Cocks and Moller found that almost one third of medicines were purchased for "personal well being" and "cultural purpose", like warding off evil spirits and to bring good luck [59].

However, clear-cut distinctions between those remedies that fall in the realm of culture and those falling into that of life-science must be treated with caution. There are epistemological similarities and differences between indigenous and biomedical notions of herbal medicine. Ashforth explains this issue vividly by illustrating how from the emic point of view, *muthi *may be activated by spirits, may be applied from spatial distance and does not necessarily have to enter the body [60]. From this perspective, *muthi *is inherently ambiguous since it can be understood either as medicine or poison according to the moral interpretation of intentions involved in its application. Without constructing a simple binary opposition between "local tradition" and "global science" it is safe to say that some interfaces and conflicts are likely to result from divergent ideas about *muthi *and its health promoting properties which are being prospected.

## The Generation of Monetary Value

With the advent of market-exchange medicinal plants, the knowledge attached to them is not only valued for healing but also for commercial purpose. African medicinal plants have entered the speculative marketplace of biocapital. The importance of investment in the "high growth" sectors of biotechnology and phytomedicine have been addressed in the founding document of the New Partnership for Africa's Development (NEPAD) [61] and later been specified by the African Biosciences Initiative [62]. Particularly in South Africa, there is a substantial market for plant based medicines. Ten years ago plant species traded for medical use already made up 20.000 tons of plant material with an estimated annual value of 38 Mil. US$ [63]. The *muthi *trade is mostly based on a "hidden economy" of self-dependent plant gatherers, street vendors and healers, which forms part of income generating strategies of rural households and especially low income women [64,65]. Considered as an untapped source for economic development, the *muthi *market is difficult to approach by state institutions and pharmaceutical companies. For successful commercialization of plant based medicine on a national and global scale, property rights are deemed prerequisite. The World Trade Organization's (WTO) Agreement on Trade Related Aspects of Intellectual Property Rights (TRIPS) protects inventions of legal persons based on novelty, non-obviousness and innovation. The agreement states that member parties may exclude plants and essential biological processes from patentability but obliges them to provide for the protection of plant varieties either by patents or by the maintenance of ex-situ plant collections or a combination thereof (Art. 27/3) [66].

One of the first efforts to establish a national collection of biomedically valuable plants in South Africa dates back to the 1990s when a company called Noristan Laboratories donated a database to the University of Cape Town's Pharmacology Department. The Traditional Medicines Project (TRAMED) was subsequently criticized by an US based group called "People's Health Alliance Rejecting Medical Authoritarianism Prejudice and Conspiratorial Tyranny" (PHARMAPACT) for illegitimately disclosing local knowledge. New information and communication technologies enabled the NGO to spread the concern of a TM sell-out. Militant accusations and some media coverage around "biopiracy" culminated in the late 1990s when the South African Medicines and Medical Devices Regulatory Authority Act was authorized (MDRA 1998) [67]. For the first time all medicines, including TM had to be registered with the Medicines Control Council and could only be sold with disclosure of their ingredients. While some argued that the regulation of TM would be vital for the safety and efficacy of treatments, others maintained that TM could not be evaluated in biomedical terms. Furthermore, it was feared the new legislation served the more subtle purpose of controlling economic revenues gained from plant medicines. Sipho Mndaweni, former president of the Interim Coordinating Committee of Traditional Practitioners in South Africa (ICCTP), was one of the first to complain that "traditional healers and vendors may end up being squeezed out of the market", insisting that "we won't see a cent of the vast profits they will make, even though people will buy these goods thinking it's the same as what we do" [68]. Although submissions were directed to Parliament to claim control over the trademark Traditional Medicine, the trend since is largely driven by attempts to standardize TM in biomedical terms [69]. Internet-platforms like TRAMED have to some extent substituted *sangomas *and *inyangas *as primary source of knowledge about endemic medical plants as candidates for bioprospecting.

In fact, several Southern African plant varieties that have been previously patented in North America and Europe are now being protected under TRIPS. An exemplary case is the patenting of a purified extract from *Harpagophytum procumbens*, assigned by the German company Finkelberg, a leading producer of plant extracts for the pharmaceutical industry. The substance may be used for the preparation of medications for the treatment of asthma, inflammatory conditions of the gastro intestinal tract or diseases of the rheumatic type [70]. The healing properties of the plant that came to be known as devil's claw have been recognized by the Khoisan people of the Kalahari long before they were primarily divulged to a German colonial soldier in the late 19^th ^century. However, the originators of the medicine were neither addressed through patent negotiations nor did they benefit from the large commercial success of the drug in Germany where it turned into the third most frequently used natural drug of all. Currently, corporations like Ratiopharm or Hexal offer a 50 g extract of devil's claw in tablet form for roughly 25 Euros. According to the German Church Development Service San plant gatherers in Namibia are at best paid 30–40 cents per kilogram of dried tubers, while others are hired for no other payment than food and drink [71].

A more recent case of controversy concerns the patenting of extracts from *Pelargonium sidoides *and *Pelargonium reniforme *by the company ISO Arzneimittel. The former is predominantly found in the South Africa's Eastern Cape while the latter is endemic throughout the cape regions and KwaZuluNatal as well as to Lesotho. There is growing bioscientific evidence of the antibacterial, antifungal and antitubercular activity of plant extracts [72]. Although *Pelargomium *has been used as medicinal plant for a variety of infectious disease by Xhosa, Zulu and Basotho people for many generations, this was neither acknowledged by the new patent holders nor by South African companies which market the drug already for more than a decade without claims to intellectual property. Officials from ISO Arzneimittel still hold that they won't challenge their South African competitors. However, according to the African Centre for Biosafety, no ABS agreement has been established in the forefront of the two patents, granted in 2003 and 2005, respectively [73]. So far, local resource providers have seen little benefit of bioprospecting, not only in the case of *Pelargonium *which is commonly used to treat HIV related infections in the region.

An assessment of the overall use of biological resources in South Africa conducted by the South African Biodiversity Institute (SANBI) found that permits were mostly applied for the collection of biological resources for "research purpose". However, frequently collectors did not participate in the "drawing up" of Material Transfer Agreements or Memoranda of Understanding. About 50% of the resource suppliers reported that there was no mechanism to share benefits; the other 50% claimed to have received some incentives like immediate cash or copies of reports [74]. The above mentioned examples may indicate a failed African Renaissance since the "re-discovery" of the medicines seems to have neither added value to economic development nor led to enhancement of health care in the region. To date, there is little data available on the impact of bioprospecting on "hidden economies" in South Africa. In how far the generation of surplus and the assignment of monetary value to plant-based medicines is something new for plant gatherers and healers as well as for bioscientist still remains to be seen. The impact of biocapital on moral economies of reciprocal gift exchange and/or redistribution which might still be prevalent in rural regions has not been paid much attention either.

## The Generation of Ethical Value

We have to take cognisance of the fact that the interrelations between the different actors in a global marketplace are complex and cannot be understood merely in terms of exclusion and repression. Apart from the generation of neo-colonial-like power relations the search for commercially valuable biogenetic or biochemical resources also includes the generation of an "ethics of sustainable development" and a fragmentation of sovereignty. From the beginning of the 1990s onwards, the assignment of economic value to biological resources has come to be seen as prerequisite for effective biodiversity conservation. In deed, an ethics of biodiversity conservation through assignment of economic value to plants is as old as the idea of bio- or chemical prospecting itself [3]. From this perspective, potential profit extracted from the commercialization of rare species to be encountered in resource rich countries provides an incentive to halt the destruction of their natural habitats [75]. Resource providers also started to struggle for shares in revenues gained by multinational corporations based on local knowledge about plants. In this context, fair bioprospecting was envisaged as a tool for sustainable development and community participation (Reid et al. 1993) [76]. The International Undertaking on Plant Genetic Resources [77] that formerly declared biodiversity to be the common heritage of human kind was abandoned with the introduction of the UN Convention on Biological Diversity.

It is well know that CBD grants nation states autonomous rights over genetic resources on their territory and recognizes collective rights of indigenous communities, which must be protected by so called Access and Benefit-Sharing (ABS) agreements. Local stakeholders of a certain resource have to be informed about bioprospecting activities, must give their formal consent to it and have to be compensated if future revenues are to be achieved (Art. 8, 16, 19) [78]. In turn, local resource providers have to commit themselves to biodiversity conservation. In South Africa this framework was finally adopted by the implementation of the National Environmental Management Biodiversity Act [79]. Applicants for patents must specify if their inventions are derived from indigenous biological resources or traditional use, which means the way in which or the purpose for which an indigenous community uses the resource in question. Subsequently, fair public-private partnerships between communities, state research institutions and pharmaceutical companies are envisioned.

There have been calls to reconsider market-oriented conservation in South Africa despite negative evidence from abroad [80]. In terms of biodiversity, the country is still one of the richest on earth. However, due to the rising demand for medical plants and the shrinkage of habitats, many species are already facing local extinction. It is assumed that almost all medical plants available in urban markets in South Africa are harvested directly in their natural habitat, mostly in an unsustainable manner [63]. In order to enhance biodiversity conservation, some ecologists are calling for large scale cultivation programs [81]. Regionally there seems to be a growing harvesting pressure to meet the demand in metropolitan centers. Several authors call for an overall cooperation in conservation strategies considering the socio-economic realities of local consumers of these medicines and those who depend on the *muthi *trade for their livelihoods [82]. Associated with the new interest in medicinal plants is not only an ethics of biodiversity conservation, but also an ethics of development similarly linked to the assignment of monetary value. It seems that the development of herbal medicines for regionally prevalent diseases is less relevant for such assignment.

The MRC with its program on traditional medicines against Malaria, Tuberculosis and AIDS-related infections still draws relatively little attention from potential private investors. There is however considerable interest in drugs that target North American and European life-style diseases. The Council for Scientific and Industrial Research has been successful in attracting several companies for the development of an appetite suppressant derived from the Hoodia cactus also used by the Khoisan. In 1995, the active substance named P57 encountered in the plant was patented by the CSIR. Six years later, a license to exploit the patent was granted to the UK based company Phytopharm [83]. At the same time the San represented by the Working Group of Indigenous Minorities in Southern Africa (WIMSA), the South African San Council, and the San Institute of South Africa (SASI) claimed San ownership to the medicine. The NGOs constructed a body of delegates that represented the San "community" and promoted negotiations with the CSIR. In 2003, the parties signed a benefit-sharing agreement that grants the CSIR and its partners the exclusive right to exploit the patent but enables the San to receive a six per cent share of the financial benefits received by the research institution from its private partners. In the case of success, approximately 0.03 percent to 1.2 percent of net sales of the product are entering a trust fund shared among the San Councils of Namibia, Botswana and South Africa [84]. The drug is currently in its last phase of clinical trials before being eventually distributed as a food supplement by the UK based multinational Unilever. Rachel Wynberg, board member of the Cape Town-based NGO Biowatch conceives the ABS agreement as a "historical breakthrough" although "there is a need for a more holistic, innovative and ethical approach to biodiversity commercialization beyond the models currently touted" [83,15].

It has been claimed that unbalanced power relations between holders of local knowledge about *muthi *and scientists must be overcome to contribute to real social innovation through bioprospecting in South Africa. From this perspective, effectiveness will depend as much on a more equitable "inward stretch" to endogenous knowledge as on "outward reach" to global sources [85]. Until recently, the testing of biological activity of crude extracts and the isolation of compounds received much more attention than the interaction between plants and people, folk taxonomies, plant-related mythology, and pre-colonial plant use [86]. The extent to which an ethics based on market-principles in Post-Apartheid South Africa is able to bridge the gap between different values accorded to medicinal plants is far from being evident yet.

## Conclusion

The process of bioprospecting involves a double translation of knowledge from traditional healers about plant based medicines and spells (*muthi*) into patentable scientific knowledge about chemical compounds and back again in terms of new property regimes introduced through Access and Benefit-Sharing (ABS) agreements. In this process, herbal medicine becomes an asset which is manipulated and transformed in relation to its capacity to promote health, its market value, and its potential to construct a new ethics of development. Property rights on *muthi *become transformed through local communities, state, and corporate interests and, on the other hand, also generate new institutions, alliances and discourses. The above mentioned examples indicate a synchronicity of the exploitation of local medical resources based on clear-cut sovereign power relations and more flexible ones in which actors and institutions of different social, ethnic and organizational strata are aligned in the same network of mutual interest. Traditional healers and San communities challenge their share in the economy of values and adopt new property regimes on their own. However, under the label of empowerment and participation which adds ethical value to state institutions and multinational corporations, still lurk historic power differentials between North and South as well as between scientists, holders of local knowledge and resource providers. In the alignment of actors and actants, the South African state still holds a hegemonic position and is drawn between the revitalization of TM as part of an identity politics that makes up for the impudence towards local knowledge in the past, on the one hand, and the interest in marketable culture on the other.

The World Trade Organization (WTO) and the Agreement on Trade Related Aspects of Intellectual Property Rights (TRIPS) do not recognize collective rights based on culture. While this is inconsistent with the CBD, currently, still surprisingly little is known about how rights of ownership and control of IK are actually negotiated in everyday transactions between scientists, pharmaceutical companies, traditional healers and local communities. By following a particular *muthi *and its plant ingredients from specific rural collection or cultivation sites to the laboratory and back again, ethnographic studies would be able to identify how, in actuality, value is conceptualized among the different stakeholders, how rights to ownership are negotiated between them and what consequences this has for the promotion of a more equitable sharing of power and knowledge in the proclaimed African Renaissance. Such an analysis would certainly start with an emic view on regimes of value among plant collectors and petty traders. Given that existing studies show that women are primarily involved in plant harvesting in rural areas, it is important to disclose how gender relations relate to the "hidden" *muthi *economy. Whether evolving cultivation projects challenge the economic survival of low income women and their families or if they have a positive impact on poverty reduction remains to be seen.

Several Anthropologists pointed out that the attempt to copyright culture by particular fractions or organizations at the expense of others might contribute to the schism of communities and fuel the establishment of ethnic boundaries. The granting of rights on the basis of ethnicity uncomfortably reminds us of Apartheid ideology. In fact, the well-intentioned benefit-sharing efforts promoted by the CBD depict a static and essential image of local culture by assuming bluntly that neo-liberal property regimes could be applied universally. While the convention addresses the misappropriation of local knowledge, it also generates new power differentials. Local medical knowledge cannot be confined to clear-cut ethnic boundaries due to well-documented dynamics of diffusion and exchange between different identity groups. To date it is not quite clear if the establishment of "trust funds" will ban such dynamics. It will be of special interest to document how far ABS agreements are contributing to a revitalized codification of local knowledge alongside ethnic boundaries and/or in relation to different types of healers and their adjunct organizations. Since there are no frameworks for assuring the representativeness of healers or other stakeholders in negotiations and ABS agreements, there is also great potential for conflict between the included and the excluded parties. The discrimination between *inyangas *in terms of pure herbalists and *sangomas *in terms of spiritual practitioners may contribute to rivalries as the latter become excluded from evolving power/knowledge networks. Future research will have to pay special attention to their strategies and tactics to enclose and protect knowledge. Indeed, it is hardly known how far bioprospecting in South Africa requires healers and local plant providers to re-conceptualize values accorded to medicinal plants, particularly in the face of new property rights.

Apart from concerns with the knowledge creation process underlying patenting, it becomes increasingly important to address the ethical dimension of separating biochemically relevant information from *muthi *and the context in which it is entwined. Biovalue can only be assigned through translation, which involves the transformation of *muthi *to standardized scientific knowledge capable of being turned into intellectual property. It still remains to be addressed how value regimes, tied to locally situated social and cultural actors and actants that come with the substance(s), are concealed step by step through technology. Anthropological studies on bioprospecting in South Africa will have to consider the entanglement of the very scientific research institutions in particular cultures and societies. The institutions involved in bioprospecting are about to transform organizational structures and processes in accordance with the values assigned to the subject of study. This is likely to transform knowledge production and reproduction itself. One of the biggest challenges in this respect will be to analyze how market-oriented research transforms the very sciences of ethnobotany, ethnobiology and ethnopharmacology. How do scientists themselves mediate their new role as businessmen, on the one hand, and as ethical arbitrators in access and benefit-sharing endeavors on the other? What impact has the search for commercially valuable and ethically appropriated resources on the topics chosen or the findings obtained in the laboratory? Finally, the problem of how to share, with whom and on what basis may turn into a matter of informal decision-making based on an ethics directed by the neo-liberal market.

The extent to which the transformation of regimes of value on medicinal plants promotes, facilitates or prohibits an African Renaissance remains to be seen. One of the myths generated in its context is the value accorded to bioprospecting as technique to simultaneously enhance the health of the 'previously' disadvantaged, to reduce poverty, to help develop a "positive" African identity, and to protect the natural environment. While there are indicators for its failure, these will have to be addressed more thoroughly by empirical research to come. Ten years after the announcement of the new nation-building project the (bio)prospect for a renaissance through so-called Traditional Medicine has neither met the high expectations of its supporters nor entailed the devastating effects of its opponents.
